# Effects of Teff-Based Sourdoughs on Dough Rheology and Gluten-Free Bread Quality

**DOI:** 10.3390/foods11071012

**Published:** 2022-03-30

**Authors:** Rosen Chochkov, Daniela Savova-Stoyanova, Maria Papageorgiou, João Miguel Rocha, Velitchka Gotcheva, Angel Angelov

**Affiliations:** 1 Technology of Cereals, Feed, Bakery and Confectionery Products Department, University of Food Technologies, 26 Maritza Blvd., 4002 Plovdiv, Bulgaria; rosen4o4kov@abv.bg (R.C.); gourmetacademy@abv.bg (D.S.-S.); 2 Department of Food Science and Technology, International Hellenic University, P.O. Box 141, 57400 Thessaloniki, Greece; mariapapage@ihu.gr; 3 LEPABE—Laboratory for Process Engineering, Environment, Biotechnology and Energy, Faculty of Engineering, University of Porto, Rua Dr. Roberto Frias, 4200-465 Porto, Portugal; jmfrocha@fe.up.pt; 4 ALiCE—Associate Laboratory in Chemical Engineering, Faculty of Engineering, University of Porto, Rua Dr. Roberto Frias, 4200-465 Porto, Portugal; 5 Department of Biotechnology, University of Food Technologies, 26 Maritza Blvd., 4002 Plovdiv, Bulgaria; gotcheva_v@uft-bio.com

**Keywords:** teff, sorghum, sourdough, lactic acid bacteria, starter cultures, gluten-free bread, celiac disease, cereals, pseudocereals

## Abstract

Production of gluten-free bread (GFB) with good quality characteristics represents a technological challenge. Our study aimed to obtain nongluten bread from cereals and pseudocereals with applying single cultures of *Pediococcus acidilactici*, *Pediococcus pentosaceus* and *Enteroccocus durans* as sourdoughs. The effect of sourdoughs on the quality traits of gluten-free (GF) dough and GFB was explored. The structural and baking properties of GF dough composed of teff, rice, corn, and sorghum flours were improved by adding xanthan gum (0.6%), guar gum (1.0%) and carboxymethyl cellulose (1.0%). The tested strains reached 10^8^ cfu/g in teff flour and produced sourdoughs with a pleasant lactic aroma. The sourdough-fermented doughs were softer and more elastic compared to control dough and yielded reduced baking loss. Strain *Enterococcus durans* ensured the best baking characteristics of GF dough and the highest softness of the GFB during storage. Strain *Pediococcus pentosaceus* had the most pronounced positive effect on aroma, taste and aftertaste. Pan baking was found to be more appropriate to obtain stable shape and good-looking products. A careful starter culture selection is necessary for GFB development since a significant effect of strain specificity on dough rheology and baking characteristics was observed.

## 1. Introduction

The incidence of gluten intolerance, or so-called ‘celiac disease’ (CD), has increased significantly over the last 50 years, affecting approximately 1% of the world’s population. If untreated, it can cause a number of serious health complications. The only way to control celiac condition is to maintain a strict gluten-free diet throughout the affected person’s life [1,2,3].

In the literature, various strategies for improving the quality of nongluten dough are suggested. However, they are mostly focused on the composition of flour mixes and food additives that aim to reproduce the viscoelastic properties of the gluten network and hence increase the development of bread loaf volume [4,5,6,7]. Dairy, soy and egg ingredients have also been used to improve the quality of gluten-free bread (GFB), but with limited success [8,9,10,11]. The need to improve the nutritional profile, appearance, taste and aroma of gluten-free breads still represents a challenge for researchers [12,13,14]. The latest innovative technological approaches include the application of high hydrostatic pressure, new baking methods (Ohmic heating) and sourdough technology [15,16].

The use of sourdough is the oldest biotechnological process to leaven bakery goods and to improve bread texture, aroma, and shelf life [17,18,19]. A number of studies have shown that sourdough can be used successfully to improve the processing characteristics of gluten-free doughs [20,21,22,23]. In some cases, this effect may be attributed to the potential of lactic acid bacteria to secrete extracellular polysaccharides, which could be a beneficial alternative to conventional thickeners used to modify the viscosity, structure and stability of a wide range of gluten-free foods [24]. These extracellular polysaccharides may act as a substitute for hydrocolloids used as food additives and, therefore, the application of lactic acid bacteria could represent a cost-efficient approach to improving the rheology of gluten-free doughs. In addition, these extracellular polysaccharides have a beneficial effect on the intestinal microbiome by selectively stimulating the growth of bifidobacteria and other beneficial microorganisms [25,26,27,28,29,30,31,32].

Cereals such as oats, sorghum and teff and various pseudocereals (buckwheat, amaranth, quinoa), tubers (cassava, potatoes), legumes (chickpea, soy, pea, lupin), nuts (acorns) and oil seeds (rapeseed, sunflower, canola) are used for the production of non-traditional flour types [6,10,11,33,34,35,36,37,38,39]. In most cases, they are superior to traditional cereal-based flours in terms of protein, essential amino acids, dietary fiber and vitamins.

One of the most widely used gluten-free cereals is rice (*Oryza sativa*). It is rich in carbohydrates (75–80%) and minerals, but protein content is only 6–7%. Potassium, magnesium, phosphorus, calcium, manganese and zinc are commonly found in rice flour. However, it is poor in trace elements due to the specific process of grain milling. Compared to corn, wheat and potatoes, rice is a relatively good source of thiamine (pantothenic acid, folic acid and vitamin E [40]. Corn flour (*Zea mays*) is combined in most cases with other types of flour to make gluten-free bread. In bakery products, it contributes to obtaining a dense and moist bread crumb. Corn flour is rich in essential nutrients, such as choline (21.6 mg/100 g), folate (48 µg/100 g), vitamins B1 (0.25 mg/100 g), B5 (0.66 mg/100 g), B6 (0.37 mg/100 g) and especially in minerals—potassium (315 mg/100 g), phosphorus (272 mg/100 g) and magnesium (93 mg/100 g) [41,42,43,44,45].

Teff flour (*Eragrostis tef*) is found to have a number of valuable nutritional characteristics compared to flours from more common crops. Its starch is slowly digestible and, therefore, has a low glycemic index (GI). It has a favorable amino acid composition and does not contain gluten [46,47]. It is a good source of unsaturated fatty acids (1.66 g/100 g) and is high in minerals, especially iron (7.63 mg/100 g) and calcium (180 mg/100 g) [48]. In addition, the high content of protein (13.3 g/100 g) and dietary fiber (8 g/100 g) makes teff flour a desirable raw material for various functional foods, some of which may bear nutritional claims such as “rich in protein” and “source of fiber” [49,50]. Teff starch has a slow retrogradation tendency, which could have a potentially positive impact on the shelf-life of baked products [51]. In addition, some authors report that the addition of teff to cereal-based sourdoughs (rice and buckwheat) modified the aroma profile of the breads, increasing the fruity, toasty and cereal notes [52,53,54,55,56,57], which contributes to developing more diverse baking products with positive consumer acceptance.

Sorghum (*Sorghum bicolor* L. Moench) is the fifth largest crop in the world. Sorghum grain has a high protein content varying from 4.4 to 21.1% with an average value of 11.4% [33,58]. Sorghum grain is gluten free, high in resistant starch, a rich source of nutrients, and most importantly, contains a diverse range of bioactive phenolic compounds [59,60]. It has been proven safe for people with celiac disease, but literature on its use in gluten-free foods is relatively scarce compared to that for corn and rice.

Since none of these gluten-free crops has satisfactory bakery performance when used alone, appropriate combinations of raw materials and processing techniques should be sought to develop successful gluten-free bakery products. Therefore, the aim of the present study was to explore the application of teff-based sourdoughs in the development of gluten-free bread with improved sensory and quality characteristics.

## 2. Materials and Methods

### 2.1. Raw Materials

The flours used in this study were teff flour (Adan Village Ltd., Sungurlare, Bulgaria), rice flour (Bodpie Food Ltd., Varna, Bulgaria), corn flour (Lubeks Ltd., Asenovgrad, Bulgaria) and sorghum flour obtained by laboratory grain milling at the University of Food Technologies, Bulgaria. Compressed yeast was supplied by Lesaffre Ltd. (Sofia, Bulgaria). Guar gum (E412) and xanthan gum (E415) were supplied by Cargill (Minneapolis, MN, USA), and carboxymethyl cellulose (E466) was supplied by Dow Wolff Cellulosics GmbH (Bomlitz, Germany).

### 2.2. Flour Characterization

Crude fat, protein, moisture, fiber and starch contents in the flours were determined according to the American Association of Cereal Chemists (AACC) methods 30–10, 46–12, 44–15, 32–05 and 76–13, respectively [61]. Protein content was calculated with a protein factor of 6.25.

### 2.3. Lactic Acid Bacteria (LAB)

Three lactic acid bacteria (LAB) strains were used in this study: *Pediococcus acidilactici* 02P108 (PA), *Pediococcus pentosaceus* 12R2187 (PP) and *Enteroccocus durans* 09B374 (ED) from the culture collection of the department of biotechnology, University of Food Technologies, Plovdiv, Bulgaria. The strains originate from typical Bulgarian sourdoughs [62,63]. Starter cultures for the sourdoughs were prepared from stock cultures of each strain stored in Microbank™ (Pro Lab Diagnostics Inc., Richmond Hill, ON, CA) by cultivation in MRS broth (de Man–Rogosa–Sharpe, Merck KGaA, Darmstadt, Germany) at 37 °C for 48 h.

### 2.4. Sourdough Preparation

Teff flour was used to prepare a separate sourdough with each LAB strain. Equal weights of flour and sterile water were mixed to obtain a dough yield (DY) of 280. Each starter culture was added to a batch of teff dough at an inoculum amount of 5 log colony-forming units (CFU)/g of dough. The sourdough variants were then fermented in an incubator at 37 °C for 24 h.

### 2.5. Analyses of Sourdoughs

Active acidity (pH) was determined by a pH meter (FiveEasy FE20, Mettler-Toledo GmbH, Greifensee, Switzerland). Total titratable acidity (TTA) was determined by titration with 0.1 N NaOH to pH 8.4 and expressed as mL of NaOH/10 g of sourdough, as described in AACC 02-31 [61]. The measurements were performed in triplicate. LAB viable cell counts were determined on MRS agar plates at the beginning and the end of the sourdough fermentation. The identity of the starter cultures in each sourdough batch was confirmed by colony morphology and microscopic observations.

### 2.6. Bread Preparation

In order to select the most appropriate formulation of a nongluten dough, gluten-free flours were mixed with an equal amount of baker’s yeast and different combinations and ratios of xanthan, guar gum and carboxymethyl cellulose. The formulations of the three tested dough variants (A, B and C) are presented in Table 1.

After selecting the most appropriate dough variant, test gluten-free breads with sourdoughs and control bread with baker’s yeast were prepared. Their formulations are presented in Table 2.

Kneading was performed by a single-phase process of dough preparation to obtain a dough with a homogeneous mass and an initial temperature of 25–26 °C. The dough was left to rest for 20 min and was then divided into pieces of 230 g—for floor bread and 440 g—for pan bread. After shaping, the dough was subjected to a final fermentation at 33 °C for 60 min in a fermenting chamber (Tecnopast CRN 45-12, Novacel Rovimpex Novaledo, Italy). The dough was then baked in an electric floor oven Salva E-25 (Salva Industrial S.L.U., Lezo, Spain), preheated to a temperature of 220–230 °C, for 17–18 min for floor bread (plain bread, baked by placing the loaf directly on the floor of the oven) and 22–25 min for pan bread (the loaf is baked in a pan). After baking, the breads were allowed to cool for 3 h at room temperature [64].

### 2.7. Degree of Immersion of the Doughs

The degree of immersion of the prepared doughs was measured by an automatic penetrometer AP-4/2 (Steinmeyer Mechatronik GmbH, Dresden, Germany). Each dough was divided into pieces of 13 g and placed in the sleeve of the penetrometer, which was then placed in a thermostat at 35 ℃ for 60 min. The immersion of the calibrated body in the dough for 5 s was automatically measured and expressed in penetrometer units (PU).

### 2.8. Rheological Properties of the Doughs

The following dough characteristics were determined by a farinograph (Brabender GmbH&Co. KG, Duisburg, Germany): water absorption (%), development time (min), stability (min), degree of softening (farinograph units (FU)) and consistency (FU), with modification of AACC Method 54-21.02 [61]. Farinographic analysis is only applied for wheat flour doughs where the reference value is 500 FU. In the present study it was used for nongluten flour combinations, with which the highest values achieved for FU were much lower (Table 3). Flour samples of 300 g were analyzed using the ICC standard method 115/1 [65]. Water absorption (WA) of the formulations was first adjusted to 65%, and then the other parameters of the doughs were measured.

### 2.9. Bread Quality

#### 2.9.1. Physical Properties

The quality of the prepared breads was assessed by the following characteristics. Bread loaf volume was determined after baking and cooling the breads for 3 h at room temperature by a rapeseed displacement method [61]. The specific volume was calculated by the ratio between volume (cm^3^) and mass (g) of each sample. Bread height and diameter were measured by a caliper, and the shape stability (height/diameter) was calculated [66]. Bake loss (%) was determined following weighing each loaf before and after baking [67]. The bread loaves were wrapped in plastic bags and stored at room temperature (27 ± 2 °C) to determine the storage time (in days) until mold growth became visible [68,69].

#### 2.9.2. Crumb Elasticity

Total and plastic deformation were measured by an automatic penetrometer and expressed as penetrometric units (PU) [69]. A 4-mm thick crumb sample was cut from the bread and placed on the flat surface of the lifting table, which was raised until the upper surface of the sample lightly touched the lower end of the immersion body. The value of penetration of the immersion body in the sample after 5 s represented total deformation. The steel disk was removed and the immersion system unloaded. Then the measurement was repeated for 10 s, and the recorded value in PU represented plastic deformation. Elastic deformation was calculated as the difference between total and plastic deformation.

### 2.10. Sensory Analysis

Sensory analyses of the obtained breads were performed by a descriptive panel consisting of 25 panelists (52% women and 48% men) aged 22–60 years, who were familiar with sensory analysis of foods but not specifically trained in the evaluation of sourdough breads. The analyses were carried out according to ISO 6658:2017 [70]. The panelists were asked to score eight parameters, namely shape, crust color, crumb color, porosity, aroma, chewability, taste and aftertaste. They expressed the intensity of each attribute on a 9-point hedonic scale (9—extremely good; 1—extremely bad).

### 2.11. Statistical Analyses

All analyses were conducted in triplicate. The obtained data were subject to one-way ANOVA using XLSTAT version 2019.1.2 (AddinSoft Inc., New York, NY, USA). Comparison among least-squares means were performed by Tukey’s test; differences were considered significant at *p*-value < 0.05 [71].

## 3. Results and Discussion

In recent years, there has been an increased interest toward using nonconventional raw materials for sourdough preparation, including both fermenting matrices and starter cultures [72,73,74,75,76,77]. In this line, the benefits of nongluten flours and lactic acid bacteria as well as applied sourdough technology were studied with the goal of developing gluten-free breads with improved nutritional and quality characteristics.

### 3.1. Flour Composition

Four types of nongluten flours were selected as raw materials for gluten-free bread preparation: teff, rice, sorghum and corn. The raw materials selection was based on literature data for the cultivars’ chemical composition. Table 4 shows the detailed chemical composition (content of moisture, protein, fiber, starch and lipids, in dry-matter (DM) basis) of the nongluten flours used in this study.

The obtained data shows that protein and fiber content of teff and sorghum flour are much higher compared to the values of these parameters for rice and corn. Protein content of sorghum was 12.49%, and in teff it was 10.20%. Furthermore, fiber content was 12.39 and 9.56%, while the values for corn and rice flour were very low—3.74 and 1.47%, respectively. These results confirm that teff and sorghum are excellent sources of protein and dietary fiber as reported by other authors [48,78,79,80,81], and could, therefore, be preferred raw materials for the development of nongluten breads with additional functionalities (rich in protein and fiber). Other authors reported approximately 11–13% of protein, approximately 80% of complex carbohydrates, and 2.4–3.0% of fat for teff [34,82]. Sorghum and teff are also generally characterized by good technological properties [83]. Apart from fiber, teff is also an excellent source of iron and contains far more calcium, potassium and other essential minerals than other grains [34,84,85,86,87,88,89]. Except for rice flour (0.59% fat), the other three studied flours contained relatively high amounts of crude fat (3.09–4.54%). It is important to point out that teff contains fat that is not easily oxidized—which results in a longer shelf-life of teff flour compared to other nongluten flours [72]. Our analyses showed that starch content was of similar levels for teff, sorghum and corn—viz., 74.5, 72.2 and 73.4%, respectively, while starch in rice flour amounted to 84.7%, which was more than 10% higher compared to the other nongluten flours. Other authors report similar results for starch content of the analyzed raw materials [72,73,81,90,91].

### 3.2. Formulation of the Control Gluten-Free Dough Matrix

To develop a gluten-free bread with good nutritional and quality characteristics, the initial step of the study was to formulate a flour blend based on the functional chemical composition as well as the technological characteristics of nongluten cereals and pseudocereals. Based on the available literature data and preliminary trials, four types of flour were selected to formulate the bread matrix—teff, rice, sorghum and corn flour.

A technological challenge with developing nongluten bakery products is that the doughs do not have an adequate structure consistency (elasticity) to retain the gas formed during fermentation. Therefore, it is necessary to use other ingredients/food additives such as proteins, starches, gums and hydrocolloids with water-binding and structure-building properties, which are able to compensate for the lack of gluten in the dough [92].

The appropriate combination of structure-forming additives and their amounts depends on the type of the nongluten flour or the specific flour combination, since these have a significant effect on the quality characteristics of the baked products. Ćuric et al. (2007) [93] supplemented a nongluten flour mix (rice flour, corn starch) with 1, 2 and 3% of xanthan, guar gum, pectin or cellulose as stabilizing additives toward the improvement of the structure of the obtained gluten-free bread and found that 3% guar gum had the best structure-forming effect.

In the present study, different combinations of xanthan, guar gum and carboxymethyl cellulose at different ratios to the nongluten flour base were tested aiming to find the most efficient gluten-replacement strategy (Materials and Methods, Section 2.6). Dough and baking characteristics of the three formulations are presented in Table 5 and Table 6.

The obtained results show that Variant B yielded a significantly higher specific volume of both floor bread (1.40 cm^3^/g) and pan bread (1.85 cm^3^/g) compared to Variants A and C (Table 6). These values were positively correlated with the highest values of the degree of immersion before and after fermentation of Variant B (Table 6) and with low bake loss—that is, 20.87% for floor bread and 17.72% for pan bread of dough variant B (Table 6). Variant C had a significantly lower shape stability (height/diameter = 0.36) compared to Variants A and B, which did not differ in this parameter. These results clearly show that the higher hydration of the nongluten dough enables a more active fermentation, but the appropriate combination of structure-forming additives is of key importance for the baking characteristics of the nongluten breads. Based on the obtained results, Variant B was selected as the control nongluten dough formulation for the next steps of the study. In terms of baking method, pan breads showed better baking characteristics, and to commercialize such nongluten formulations, pan baking would be more appropriate to obtain good-looking products.

### 3.3. Sourdough Fermentation

The sourdoughs applied for leavening of the main gluten-free flour mix were prepared only from teff flour with the addition of the three different cultures of lactic acid bacteria. The kinetics of sourdough fermentation was monitored by the changes in pH, TTA and the total viable counts of the respective starter culture. Results from these analyses are presented in Table 7.

The obtained results clearly indicate a good capacity of the three tested strains to ferment teff flour, while increasing their biomass and producing organic acids, which resulted in sourdoughs with a pleasant lactic acid aroma. Indeed, the genera *Pediococcus* and *Enterococcus* are homofermentative or homolactic bacteria, thus releasing solely lactic acid from fermentation—and known as hexose fermentation pathway or Embden–Meyerholf–Parnas (EMP) pathway. Data analysis showed no statistical differences in the yielded biomass concentration at the end of the fermentations among the three tested LAB strains (8.72–8.83 log CFU/g). The lowest pH value (3.88) was reached in the sourdough with strain *Pediococcus pentosaceus* 12R2187, but it was not significantly different compared to the other two sourdoughs. One of the strains used to prepare sourdough was of *Enteroccocus durans* species, which is not commonly found in sourdoughs. It was interesting to observe that this strain yielded a final TTA value of 18.4, which was significantly higher compared to the other two LAB strains and indicated the highest capacity of strain *Enteroccocus durans* 09B374 to produce organic acids.

### 3.4. Development of Gluten-Free Bread with Sourdough

#### 3.4.1. Degree of Immersion in the Nongluten Sourdoughs

**Figure 1:**
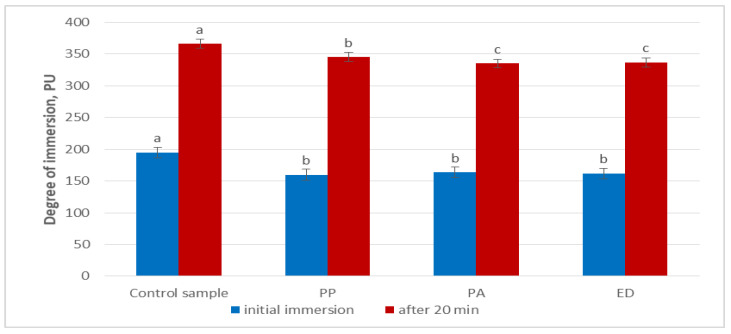
Degree of immersion in nongluten doughs prepared with the addition of sourdoughs with single cultures lactic acid bacteria. Note: PU—penetrometric units; PA—sample with *Pediococcus acidilactici* 02P108; PP—sample with *Pediococcus pentosaceus* 12R2187; ED—sample with *Enteroccocus durans* 09B374. Different small letters for each time measurement indicate significant difference between mean values (*p* < 0.01).

Three nongluten doughs were prepared by using as a matrix the preselected dough variant B (Section 3.2) without yeast and by adding equal amounts of teff sourdoughs prepared with the three selected lactic acid bacteria strains—*Pediococcus acidilactici* 02P108, *Pediococcus pentosaceus* 12R2187 and *Enteroccocus durans* 09B374. Dough variant B with yeast was used as the control. All nongluten doughs were kneaded according to the established methodology (Section 2.6). The results from analyzing the degree of immersion in the nongluten doughs are presented in Figure 1.

The initial degree of immersion of the control dough was significantly higher (195 PU) (*p* < 0.01) compared to each of the three samples with added sourdoughs (160–164 PU). However, the difference between these three samples was not significant. After 20 min of fermentation, the degree of immersion of the control sample increased by 87.7%, and for all sourdough-fermented doughs, the values more than doubled (335–345 PU). However, for all three sourdough-fermented samples, the degree of immersion was significantly lower than that of the control sample (366 PU). These results indicate that the sourdough-fermented doughs are more resistant and less elastic compared to the control dough fermented by baker’s yeast (*Saccharomyces cerevisiae*). These observations differ from the results reported by Wolter et al. (2014) [94], where sourdough addition led to decreased dough strength resulting in softer dough. Other authors also confirm that sourdough fermentation increased the elasticity and reduced the stiffness of doughs [95]. However, these teams studied wheat-based doughs, while with nongluten doughs this effect is the opposite, as observed in our study. The reduced elasticity could be attributed to the nongluten flour composition and the specific fermentation capacity of the tested strains. Other studies on the rheological properties of gluten-free sourdoughs prepared with lactic acid bacteria also found that the addition of sourdough reduced the elasticity of the dough and improved the dough strength [69,96,97].

In our study, after 20 min of fermentation, strain PP produced a significantly softer dough compared to the other two strains. Such findings confirmed that the reduced elasticity was attributed to the LAB fermentation and that strain specificity has an effect on nongluten dough stability.

#### 3.4.2. Effect of Sourdoughs on the Rheological Characteristics of Nongluten Dough

The results from the rheological analysis of the nongluten doughs obtained with the addition of sourdoughs with different lactic acid bacteria are depicted in Table 3.

A farinograph is a useful tool for the determination of the optimal water content for dough preparation. In addition, it provides information about dough stability and dough development time [98]. In general, the water-absorbing capacity of flour depends on the protein content, the particle size, the amount of starchy grains with impaired integrity and some other factors. In the present study, the addition of hydrocolloids also contributed to the water absorption and to the improved and rheological properties. Our preliminary experiment with different WA values (55%, 65% and 70%) of nongluten flour basis showed that 65% provided best dough consistency as well as best specific volume and porosity of the bread (data not shown). Based on these findings, the WA of the formulations in the current study was first adjusted to 65%, and then the other parameters of the doughs were measured.

The consistency of the control sample was 20.69% higher than that of the experimental samples. No significant differences were observed in the consistency (290 FU) of the samples with added sourdoughs. Significant differences between the control and the sourdough-leavened doughs were also observed for the other tested parameters: dough development time (DDT), stability and relaxation of the dough. The DDT of the sourdough-added samples was reduced by 3 times (2 min) for sample PP and by 4 times (1.5 min) for samples PA and ED. These results are in contrast to the observations of Tafti et al. (2013) [99] who found no effect of spray-dried sourdough addition on wheat dough development time. Again, the difference might be attributed to the different kind of dough matrix.

In our study, the control sample had significantly higher stability (10 min) than the sourdough-fermented samples. However, the values of 6.5–7.5 min also indicated good stability of the doughs obtained with sourdough addition and with dough samples PP and PA showing significantly better effect compared to the sample ED. The degree of dough softening of the control sample was 10 FU, while the sourdough-fermented doughs showed a significantly higher value (30 FU), which is still not too high for this parameter and was the same for the three test samples. Tafti et al. (2013) [99] also observed that the degree of softening significantly increased with an increase in the sourdough level, whereas dough stability was significantly reduced. The results obtained in our study indicate that the use of sourdoughs requires some additional matrix optimization to achieve the same dough rheology as the yeast-fermented dough.

#### 3.4.3. Quality Assessment of Gluten-Free Breads Leavened with Sourdough

To assess the baking characteristics of the gluten-free breads with added sourdough, two types of bread—floor and pan bread—were prepared from each sourdough-leavened bread variety. Fermentation and baking of all dough samples were carried out under equal conditions, according to the adopted technology. Results from the quality assessment of their baking characteristics are presented in Table 8.

The specific volumes of the floor bread samples prepared with strains PA and ED were significantly higher compared to the control and sample PP, with strain *Enteroccocus durans* yielding the highest value (1.70 cm^3^/g). Comparison of pan bread samples showed that samples PP and PA had significantly lower (20% for sample PP) specific volumes than the control sample and sample ED. These results indicate that strain specificity is important in terms of the generated specific volume of nongluten breads, and in our study, strain *Enteroccocus durans* gave the best performance in leavening the nongluten dough.

Literature data on the effect of LAB on the specific volume of gluten-free breads are diverse. According to some authors, the addition of sourdough to GF breads does not have a significant influence on the specific volume [100,101]. Cappa et al. (2016) [17] reported that sourdoughs have been effective in improving bread volume and softness, which confirms the positive effects observed in our experiments. Other studies also showed that sourdough gluten-free bread had a higher specific volume and was less firm than GF bread fermented with baker’s yeast alone [102,103].

In terms of shape stability (height/diameter ratio, H/D), strain ED gave a significantly higher value of 0.48 compared to the other three tested doughs. Similar to this result, Falade et al. (2014) [104] reported that the addition of lactic acid bacteria increased the bread height after baking.

It is interesting to note that all three LAB-leavened samples had significantly lower baking loss compared to the yeast-leavened control sample, with the lowest value (15.65%) among floor breads observed for sample ED, and the lowest among pan breads (12.25%) observed for sample PA. This positive effect could be attributed to the organic acids produced by the bacteria, which strengthen the structure of the gels in the gluten-free doughs. Therefore, the gas retention in the bread is greater. Our observations confirm the findings of Wolter et al. (2014) [94] and indicate that the application of sourdoughs does affect the baking characteristics of the gluten-free bread, mostly by a significantly pronounced reduction in baking loss. A strain-specific effect on the analyzed parameters was also observed, and this effect differed with respect to the type of bread—i.e., floor or pan.

#### 3.4.4. Sensory Profile of Nongluten Breads with Sourdough

**Figure 2:**
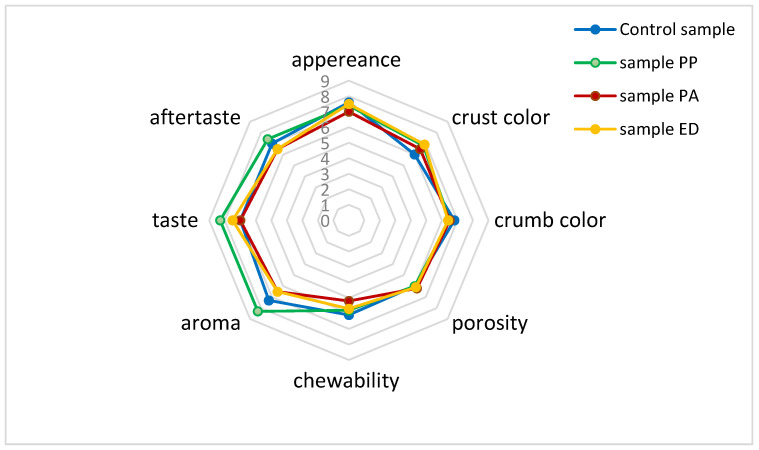
Sensory profile of nongluten pan breads with sourdough. Note: *Pediococcus acidilactici* 02P108 (PA); *Pediococcus pentosaceus* 12R2187 (PP); *Enteroccocus durans* 09B374 (ED).

In general, the use of LAB-inoculated sourdough can improve the quality of bread with regards to various characteristics such as taste, staleness, odor, chewability, softness, moisture content, pH, acidity and texture [105]. In the present study, the gluten-free breads leavened with sourdoughs differed in appearance from the control mainly by a better color of the crust (Figure 2), especially of sample ED. The crust of the three samples was thin, smooth and soft.

Generally, all three strains reached biomass content at the level of 10^8^ CFU/g, but the differences in the fermenting capacity showed variations in acid production as well as the composition of the produced organic acids. These differences resulted in variations in the sensory characteristics of the obtained nongluten breads (Section 3.4.4).

The ratio between lactic and acetic acids s an important factor affecting the aroma of bread [106], and its influenced by the fermenting microorganisms, the fermentation temperature and the type of flour or flours [107]. In the present study, the aroma, taste and aftertaste were found pleasant for all samples prepared from the nongluten flour mix. However, these characteristics were most pronounced in the sample with strain *Pediococcus pentosaceus* 12R2187 (PP), with a significant difference compared to the other samples.

According to Moore et al. (2008) [100], acidification during sourdough fermentation increases polysaccharide swelling that can partially replace gluten and improve the structure of gluten-free bread. In our study, the development of bread porosity in the sourdough-fermented bread samples did not differ from the control sample. The middle of all samples was soft and slightly moist, and slight sticking to the teeth was observed while chewing. Indeed, only sample PA gave a significant difference compared to control regarding this parameter. Data from the sensory analysis showed positive acceptance of the prepared nongluten breads. Some sensory characteristics were significantly influenced by the LAB strain specificity, with the most pronounced positive effect shown by strain *Pediococcus pentosaceus* 12R2187 (PP).

#### 3.4.5. Shelf-Life Estimation of Nongluten Bread with Sourdough

**Figure 3:**
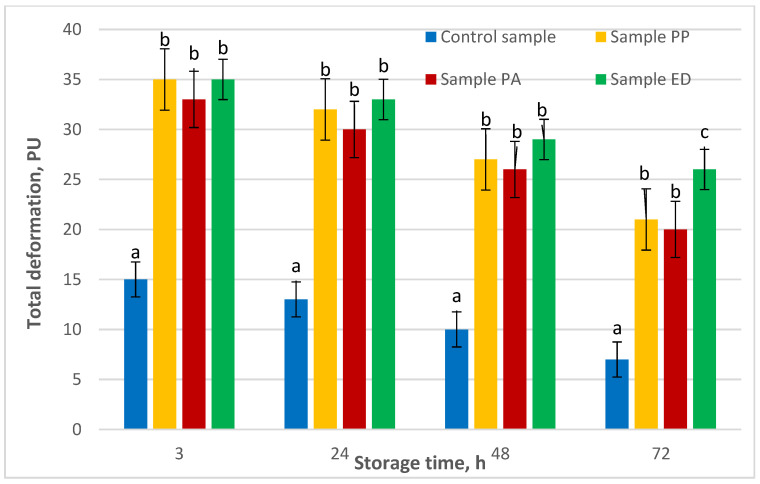
Effect of the different LAB strains on the total deformation of nongluten bread. Note: PU—penetrometric units; PA—sample with *Pediococcus acidilactici* 02P108; PP—sample with *Pediococcus pentosaceus* 12R2187; ED—sample with *Enteroccocus durans* 09B374. Different small letters for each time measurement indicate significant difference between mean values (*p* < 0.01).

Bourne (1978) [108] described the use of instrumental texture profile analysis extensively, using force, deformation, and work measurement to determine the texture parameters for hardness, fracturability, cohesiveness, adhesiveness, springiness, gumminess and chewiness. In the present research, estimation of the shelf life of the prepared gluten-free pan breads with sourdoughs was based on the time of occurrence of mold growth, and the analysis of the deformation characteristics—viz., total, elastic and plastic deformation—were measured by an automatic penetrometer of the bread crumb. Pan breads were selected to explore deformation characteristics since this method of baking showed better baking characteristics of the nongluten formulations. The total deformation of the breads leavened with lactic acid bacteria (33–35 PU) was more than two times higher than that of the yeast-leavened control sample (15 PU) (Figure 3). This trend was generally preserved until the end of the experiment (72 h), while total deformation gradually decreased for all samples.

It is interesting to note that the total deformation differences between the three samples with sourdoughs were not significant during the 48 h of fermentation, and only at the end of the process did samples PP and PA have a significantly lower total deformation (21 and 20 PU, respectively) compared to sample ED (26 PU).

The total deformation of the control sample was reduced by 53% for 72 h, while the average reduction for the breads with sourdough addition was 25%, with the largest decrease observed for sample PP (40%), and the lowest for sample ED (26%). These results clearly show that the application of LAB improves the softness of bread throughout storage time, with the strongest effect observed for strain *Enteroccocus durans* 09B374 (ED). In a study on the application of sourdough *Lactobacillus* strains to obtain gluten-free bread, Di Cagno et al. (2008) [103] also found that the addition of lactic acid bacteria resulted in lower hardness of gluten-free bread crumb during storage.

**Figure 4:**
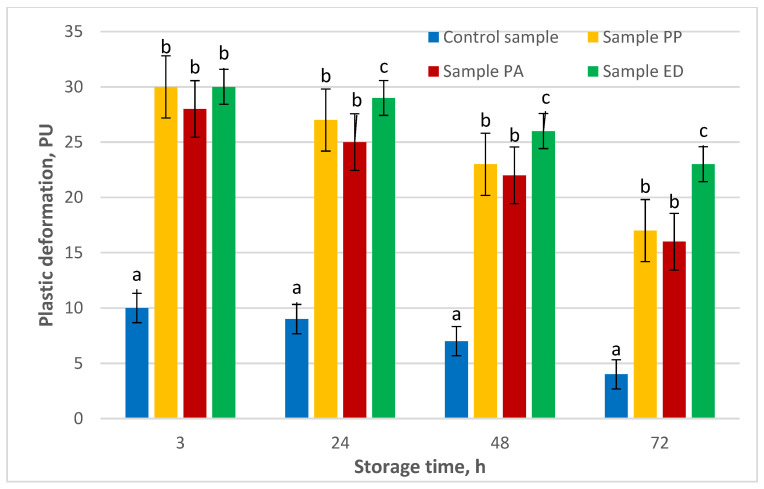
Effect of lactic acid bacteria on the plastic deformation of nongluten bread. Note: PU—penetrometric units; PA—sample with *Pediococcus acidilactici* 02P108; PP—sample with *Pediococcus pentosaceus* 12R2187; ED—sample with *Enteroccocus durans* 09B374. Different small letters for each time measurement indicate significant difference between mean values (*p* < 0.01).

Results from analyzing the plastic deformation of sourdough-leavened nongluten breads are displayed in Figure 4.

The plastic deformation of the sourdough-leavened breads was considerably greater (around threefold more) compared to the control, and it gradually decreased until the end of the experiment. The crumb of sample ED retained its plasticity to the greatest extent, which was most pronounced at the end of the 72 h test. Plasticity of this sample decreased by 25% between the third and the 72nd hour compared to a 60% reduction observed in the control. For the other two LAB-leavened samples, plasticity decreased by an average of 44%. After 24 h storage, the plastic deformation of sample ED became significantly higher compared to sample PA, and this trend was maintained until the end of the experiment, when plastic deformation of sample ED was significantly higher than sample PP as well. The difference between samples PP and PA was not significant during the course of the entire duration of the experiment. These results indicate that the microbial strain specificity affects the plastic deformation of nongluten breads during storage, and in this study strain *Enteroccocus durans* 09B374 (ED) provided the highest softness of the nongluten bread in terms of total and plastic deformation.

**Figure 5:**
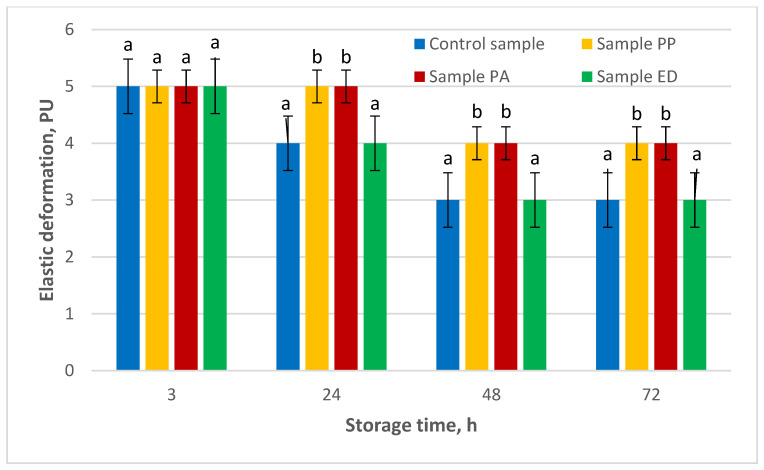
Effect of lactic acid bacteria on the elastic deformation of nongluten bread. Note: PU—penetrometric units; PA—sample with *Pediococcus acidilactici* 02P108; PP—sample with *Pediococcus pentosaceus* 12R2187; ED—sample with *Enteroccocus durans* 09B374. Different small letters for each time measurement indicate significant difference between mean values (*p* < 0.01).

The third analyzed shelf-life parameter for the nongluten breads prepared with the additions of sourdoughs was elastic deformation (Figure 5).

Elastic deformation measurement at 3 h of storage showed the same average values (5 PU) for all tested samples. The relaxation capacity of the gluten-free bread samples PP and PA did not change after 24 h and remained at 4 PU at 48 and 72 h, while sample ED behaved similar to the control sample and also did not change between 48 and 72 h. Results for samples PP and PA were significantly different (*p* < 0.01) compared to control and sample ED, which indicates the effect of strain specificity on the elastic properties during storage.

Many other studies also demonstrated that sourdough gluten-free bread was less firm than gluten-free bread leavened with baker’s yeast alone [101,103,109,110]. Shelf-life rheological tests made by Moore et al. (2008) [101], however, showed that the addition of sourdough to a gluten-free mix led to increased firmness and elasticity overtime, which indicated that a LAB strain could be used to produce gluten-free bread with increased quality and shelf life. A study of Moroni et al. (2009) [24] also showed that the use of sourdough in nongluten bread development had positive effects on the crumb structure. Staling was delayed and longer shelf-life was achieved. These effects are mostly associated with the production of lactic and acetic acids, as well as exopolysaccharides during fermentation with lactic acid bacteria. In addition, the use of LAB-inoculated sourdoughs in bread preparation may contribute antifungal properties, thereby increasing the shelf-life of the bread even at a reduced salt content [111].

## 4. Conclusions

The selected combination of nongluten flours and additives was adequate for obtaining nongluten bread with good quality characteristics. The three tested LAB strains demonstrated good capacity to ferment teff flour into sourdough, reaching different levels of acidification. The application of teff-based sourdoughs had a positive effect on various technological and sensory characteristics of the nongluten doughs and breads. The sourdough-fermented doughs were softer and more elastic compared to the control dough fermented by baker’s yeast. The application of sourdoughs resulted in a significantly pronounced reduction of baking loss. A strain-specific effect on the analyzed quality parameters was observed, and this effect also differed with respect to the bread type—floor or pan bread. Pan baking resulted in better bread characteristics and proved to be more appropriate for commercialization of nongluten bread formulations.

The obtained nongluten breads had positive sensory acceptance. Strain specificity had a significant effect on some sensory characteristics of the products and on bread softness during storage.

The study demonstrated that the application of sourdoughs in the nongluten flour matrix is a successful approach for gluten-free bread development. Strain specificity is significant for dough rheology and the baking characteristics, and it is, therefore, important to perform a careful starter culture selection.

## Figures and Tables

**Table 1:** Composition of gluten-free bread formulations.

Ingredients		Variants		
		A	B	C
		Quantity, %		
Teff flour	Gluten-free flour base	40	40	40
Rice flour		40	40	40
Sorghum flour		10	10	10
Corn flour		10	10	10
Other ingredients, g/100 g Gluten-free flour base				
Water		65	65	65
Baker’s yeast		3.0	3.0	3.0
Salt		1.5	1.5	1.5
Xanthan gum		1.0	0.6	0.6
Guar gum		1.0	1.0	1.0
Carboxymethyl cellulose (CMC)			1.0	3.0

**Table 2:** Composition of gluten-free breads with sourdoughs and control bread.

Ingredients		Control Sample	Sample PP	Sample PA	Sample ED
		Quantity, %			
Teff flour	Gluten-free flour base	40	32.8		
Rice flour		40	40.0		
Sorghum flour		10	10.0		
Corn flour		10	10.0		
Other ingredients, g/100 g Gluten-free flour base					
Water		65	52.4		
Baker’s yeast		3.0	-		
Salt		1.5	1.5		
Xanthan gum		0.6	0.6		
Guar gum		1.0	1.0		
Carboxymethyl cellulose (CMC)		1.0	1.0		
Sourdough (Teff flour + Water + LAB)			21.5		

**Table 3:** Effect of sourdoughs on the rheological characteristics of nongluten dough.

Samples	Rheological Characteristics				
	Water Absorption, %	Consistency, FU	DDT, min	Stability, min	Degree of Softening, FU
Control sample	65 ± 3.56 ^a^	350 ± 1.41 ^a^	6.0 ± 0.82 ^a^	10.0 ± 0.82 ^a^	10 ± 0.82 ^a^
Sample PP	65 ± 5.65 ^a^	290 ± 1.83 ^b^	2.0 ± 0.82 ^b^	7.5 ± 0.08 ^b^	30 ± 0.82 ^b^
Sample PA	65 ± 0.82 ^a^	290 ± 2.58 ^b^	1.5 ± 0.22 ^b^	7.5 ± 0.08 ^b^	30 ± 0.82 ^b^
Sample ED	65 ± 5.72 ^a^	290 ± 0.82 ^b^	1.5 ± 0.08 ^b^	6.5 ± 0.29 ^c^	30 ± 0.82 ^b^

Mean values with different letter in superscript within the same column differ significantly (*p* < 0.05). Note: *Pediococcus acidilactici* 02P108 (PA); *Pediococcus pentosaceus* 12R2187 (PP); *Enteroccocus durans* 09B374 (ED). DDT, dough development time.

**Table 4:** Chemical composition of the nongluten flours.

Flour	Moisture Content, %	Protein Content, % (d.m.)	Fiber Content, % (d.m.)	Crude Fat, % (d.m.)	Starch Content, % (d.m.)
Teff	10.18 ± 0.16 ^a^	10.20 ± 0.13 ^a^	12.39 ± 0.13 ^a^	3.09 ± 0.16 ^a^	74.5 ± 0.80 ^a^
Rice	10.00 ± 0.14 ^a^	6.99 ± 0.10 ^b^	1.47 ± 0.16 ^b^	0.59 ± 0.09 ^b^	84.7 ± 1.07 ^b^
Sorghum	11.04 ± 0.24 ^b^	12.49 ± 0.46 ^c^	9.56 ± 0.08 ^c^	4.54 ± 0.12 ^c^	72.2 ± 0.92 ^c^
Corn	9.05 ± 0.16 ^c^	4.20 ± 0.09 ^d^	3.74 ± 0.12 ^d^	3.73 ± 0.08 ^d^	73.4 ± 0.77 ^d^

Mean values with different letter in superscript within the same column differ significantly (*p* < 0.05).

**Table 5:** Fermentation characteristics of nongluten dough formulations.

Dough Variant	Preliminary Degree of Immersion, PU	Degree of Immersion After 20 min, PU
A	70 ± 1.63 ^a^	117 ± 3.74 ^a^
B	195 ± 4.55 ^b^	366 ± 0.82 ^b^
C	118 ± 1.63 ^c^	190 ± 3.46 ^c^

Variant A—xanthan 1.0% and guar gum 1.0%; Variant B—xanthan 0.6%, guar gum 1.0% and CMC 1.0%; Variant C—xanthan 0.6%, guar gum 1.0% and CMC 3.0%. Mean values with different letter in superscript within the same column differ significantly (*p* < 0.05).

**Table 6:** Baking characteristics of nongluten dough formulations.

Dough Variant	Height/Diameter	Specific Volume, cm^3^/g		Bake Loss, %	
	Floor Bread	Floor Bread	Pan Bread	Floor Bread	Pan Bread
A	0.45 ± 0.03 ^a^	0.90 ± 0.08 ^a^	1.12 ± 0.09 ^a^	22.61 ± 0.07 ^a^	20.68 ± 0.09 ^a^
B	0.45 ± 0.02 ^a^	1.40 ± 0.06 ^b^	1.85 ± 0.09 ^b^	20.87 ± 0.11 ^b^	17.72 ± 0.05 ^b^
C	0.36 ± 0.01 ^b^	0.95 ± 0.05 ^a^	0.88 ± 0.09 ^c^	21.30 ± 0.64 ^b^	16.14 ± 0.67 ^c^

Variant A—xanthan 1.0% and guar gum 1.0%; Variant B—xanthan 0.6%, guar gum 1.0% and CMC 1.0%; Variant C—xanthan 0.6%, guar gum 1.0% and CMC 3.0%. Mean values with different letter in superscript within the same column differ significantly (*p* < 0.05).

**Table 7:** Fermentation kinetics of teff sourdoughs.

Strain/Time	*Pediococcus acidilactici* 02P108 (PA)			*Pediococcus pentosaceus* 12R2187 (PP)			*Enteroccocus durans* 09B374 (ED)		
	pH	TTA	log CFU/g	pH	TTA	log CFU/g	pH	TTA	log CFU/g
0 h	5.92 ± 0.06	4.2 ± 0,25	3.00 ± 0.11	5.92 ± 0.18	4.2 ± 0.29	2.95 ± 0.19	5.92 ± 0.18	4.2 ± 0.40	2.90 ± 0.57
4 h	5.48 ± 0.10	4.6 ± 0.26	3.08 ± 0.21	5.40 ± 0.23	4.7 ± 0.31	3.04 ± 0.18	5.26 ± 0.14	4.6 ± 0.31	2.95 ± 0.38
8 h	5.26 ± 0.10	5.8 ± 0.13	4.63 ± 0.64	5.32 ± 0.18	5.6 ± 0.26	4.62 ± 0.31	5.12 ± 0.36	5.9 ± 0.26	4.71 ± 0.59
12 h	4.88 ± 0.14	9.6 ± 0.31	6.26 ± 0.35	5.02 ± 0.28	8.8 ± 0.25	6.75 ± 0.21	4.65 ± 0.26	10.0 ± 0.26	6.86 ± 0.67
16 h	4.32 ± 0.15	13.2 ± 0.25	6.79 ± 0.49	4.64 ± 0.19	13.4 ± 0.38	6.95 ± 0.47	4.28 ± 0.35	15.3 ± 0.29	7.00 ± 0.71
20 h	4.12 ± 0.10	14.9 ± 0.45	8.15 ± 0.24	4.04 ± 0.38	14.2 ± 0.28	8.28 ± 0.26	4.15 ± 0.12	17.1 ± 0.31	8.53 ± 0.49
24 h	4.02 ± 0.10	15.2 ± 0.26	8.72 ± 0.59	3.88 ± 0.27	14.6 ± 0.33	8.81 ± 0.51	4.10 ± 0.21	18.4 ± 0.34	8.83 ± 0.27
	a	a	a	a	a	a	a	b	a

Mean values of the same parameters with different letter within the same row differ significantly (*p* < 0.05).

**Table 8:** Baking characteristics of nongluten breads with sourdough.

Dough Samples	Specific Volume, cm^3^/g		Height/ Diameter	Baking Loss, %	
	Floor Bread	Pan Bread	Floor Bread	Floor Bread	Pan Bread
Control sample	1.49 ± 0.01 ^a^	1.55 ± 0.01 ^a^	0.36 ± 0.01 ^a^	20.86 ± 0.06 ^a^	17.72 ± 0.05 ^a^
Sample PP	1.55 ± 0.06 ^a^	1.24 ± 0.03 ^b^	0.30 ± 0.04 ^a^	18.90 ± 0.06 ^b^	13.02 ± 0.03 ^b^
Sample PA	1.59 ± 0.01 ^b^	1.46 ± 0.01 ^c^	0.36 ± 0.03 ^a^	16.70 ± 0.01 ^c^	12.25 ± 0.17 ^c^
Sample ED	1.70 ± 0.03 ^c^	1.56 ± 0.02 ^a^	0.48 ± 0.04 ^b^	15.65 ± 0.04 ^d^	12.59 ± 0.07 ^d^

Mean values with different letter in superscript within the same column differ significantly (*p* < 0.05). Note: *Pediococcus acidilactici* 02P108 (PA); *Pediococcus pentosaceus* 12R2187 (PP); *Enteroccocus durans* 09B374 (ED).

## Data Availability

Not applicable.

## Abbreviations

AACC, American Association of Cereal Chemists; CD, celiac disease; CFU, colony-forming units; C-GFB, control gluten-free bread; CMC, carboxymethyl cellulose; DDT, dough development time; DM, dry-matter; DY, dough yield; ED, *Enteroccocus durans* 09B374; EMP, Embden-Meyerholf-Parnas; FU, farinograph units; GFB, gluten-free bread; GI, glycemic index; H/D, Height/Diameter ratio; LAB, lactic acid bacteria; MRS, de Man-Rogosa-Sharpe; PA, *Pediococcus acidilactici* 02P108; PP, *Pediococcus pentosaceus* 12R2187; PU, penetrometer units; TTA, total titratable acidity.

## References

[1] Cappelli A., Oliva N., Cini E. (2020). A systematic review of gluten-free dough and bread: Dough rheology, bread characteristics, and improvement strategies. Appl. Sci..

[2] Novotni D., Gänzle M., Rocha J.M., Galanakis C.M. (2020). Composition and Activity of Microbiota in Sourdough and Their Effect on Bread Quality and Safety. Trends in Wheat and Bread Making.

[3] Rocha J.M. (2011). Microbiological and Lipid Profiles of Broa: Contributions for the Characterization of a Traditional Portuguese Bread. Ph.D. Thesis.

[4] Galle S., Schwab C., dal Bello F., Coffey A., Ganzle M.G., Arendt E.K.K. (2012). Influence of in situ synthesized exopolysaccharides on the quality of gluten-free sorghum sourdough bread. Int. J. Food Microbiol..

[5] Padalino L., Conte A., del Nobile A.M. (2016). Overview on the general approaches to improve gluten-free pasta and bread. Foods.

[6] Skendi A., Papageorgiou M., Varzakas T. (2021). High protein substitutes for gluten in gluten-free bread. Foods.

[7] Culetu A., Duta D.E., Papageorgiou M., Varzakas T. (2021). The role of hydrocolloids in gluten-free bread and pasta; rheology, characteristics, staling and glycemic index. Foods.

[8] Guimaraes R.M., Pimentel T.C., de Rezende T.A.M., Silva J.S., Falcao H.G., Ida E.I., Egea M.B. (2019). Gluten-free bread: Effect of soy and corn co-products on the quality parameters. Eur. Food Res. Technol..

[9] Han A., Romero H.M., Nishijima N., Ichimura T., Handa A., Xu C., Zhang Y. (2019). Effect of egg white solids on the rheological properties and bread making performance of gluten-free batter. Food Hydrocoll..

[10] Pico J., Reguilón M.P., Bernal J., Manuel Gómez M. (2019). Effect of rice, pea, egg white and whey proteins on crust quality of rice flour-corn starch based gluten-free breads. J. Cereal Sci..

[11] Taghdir M., Mazloomi S.M., Honar N., Sepandi M., Ashourpour M., Salehi M. (2017). Effect of soy flour on nutritional, physicochemical, and sensory characteristics of gluten-free bread. Food Sci. Nutr..

[12] Păcularu-Burada B., Turturică M., Rocha J.M., Bahrim G.-E. (2021). Statistical approach to potentially enhance the postbiotication of gluten-free sourdough. Appl. Sci..

[13] Păcularu-Burada B., Georgescu L.A., Vasile M.A., Rocha J.M., Bahrim G.-E. (2020). Selection of wild lactic acid bacteria strains as promoters of postbiotics in gluten-free sourdoughs. Microorganisms.

[14] Bartkiene E., Lele V., Ruzauskas M., Mayrhofer S., Domig K., Starkute V., Zavistanaviciute P., Bartkevics V., Pugajeva I., Klupsaite D. (2020). Lactic acid bacteria isolation from spontaneous sourdough and their characterization including antimicrobial and antifungal properties evaluation. Microorganisms.

[15] Vallons K.J.R., Ryan L.A.M., Arendt E.K. (2011). Promoting structure formation by high pressure in gluten-free flours. LWT Food Sci. Technol..

[16] Bender D., Gratz M., Vogt S., Fauster T., Wicki B., Pichler S., Kinner M., Jäger H., Schoenlechner R. (2019). Ohmic heating—A novel approach for gluten-free bread baking. Food Bioprocess Technol..

[17] Cappa C., Lucisano M., Raineri A., Fongaro L., Foschino R., Mariotti M. (2016). Gluten-free bread: Influence of sourdough and compressed yeast on proofing and baking properties. Foods.

[18] Sun L., Li X., Zhang Y., Yang W., Ma G., Ma N., Hu Q., Pei F. (2020). A novel lactic acid bacterium for improving the quality and shelf life of whole wheat bread. Food Control.

[19] Teleky B.E., Martau G.A., Vodnar D.C. (2020). Physicochemical effects of *Lactobacillus plantarum* and *Lactobacillus casei* cocultures on soy-wheat flour dough fermentation. Foods.

[20] Moore M.M., Heinbockel M., Dockery P., Ulmer H.M., Arendt E.K. (2006). Network formation in gluten-free bread with application of transglutaminase. Cereal Chem..

[21] Moore M.M., Juga B., Schober T.J., Arendt E.K. (2007). Effect of lactic acid bacteria on properties of gluten-free sourdoughs, batters, and quality and ultrastructure of gluten-free bread. Cereal Chem..

[22] Ngemakwe P.H., le Roes-Hill M., Jideani V.A. (2015). Advances in gluten-free bread technology. Food Sci. Technol. Int..

[23] Matos M.E., Rosell C.M. (2015). Understanding gluten-free dough for reaching breads with physical quality and nutritional balance. J. Sci. Food Agric..

[24] Moroni A.V., dal Bello F., Arendt E.K. (2009). Sourdough in gluten-free bread-making: An ancient technology to solve a novel issue?. Food Microbiol..

[25] Di Cagno R., de Angelis M., Lavermicocca P., de Vincenzi M., Giovannini C., Faccia M., Gobbetti M. (2002). Proteolysis by sourdough lactic acid bacteria: Effects on wheat flour protein fractions and gliadin peptides involved in human cereal intolerance. Appl. Environ. Microbiol..

[26] Deora N.S., Deswal A., Mishra H.N. (2014). Alternative approaches towards gluten-free dough development: Recent trends. Food Eng. Rev..

[27] Ren Y., Linter B.R., Linforth R., Foster T.J. (2020). A comprehensive investigation of gluten free bread dough rheology, proving and baking performance and bread qualities by response surface design and principal component analysis. Food Funct..

[28] Bartkiene E., Özogul F., Rocha J.M. (2020). Bread sourdough lactic acid bacteria—Technological, antimicrobial, toxin-degrading, immune system- and faecal microbiota-modelling biological agents for the preparation of food, nutraceuticals and feed. Foods.

[29] Ağagündüz D., Yılmaz B., Şahin T.O., Güneşliol B.E., Ayten S., Russo P., Spano G., Rocha J.M., Bartkiene E., Özogul F. (2021). Dairy lactic acid bacteria and their potential function in dietetics: The food-gut-health axis. Foods.

[30] Sharma H., Özogul F., Bartkiéne E., Rocha J.M. (2021). Impact of lactic acid bacteria and their metabolites on the techno-functional properties and health benefits of fermented dairy products. Crit. Rev. Food Sci. Nutr..

[31] Skendi A., Zinoviadou K.G., Papageorgiou M., Rocha J.M. (2020). Advances on the valorisation and functionalization of by-products and wastes from cereal-based processing industry. Foods.

[32] Zokaityte E., Cernauskas D., Klupsaite D., Lele V., Starkute V., Zavistanaviciute P., Ruzauskas M., Gruzauskas R., Juodeikiene G., Rocha J.M. (2020). Bioconversion of milk permeate with selected lactic acid bacteria strains and apple by-products into beverages with antimicrobial properties and enriched with galactooligosaccharides. Microorganisms.

[33] Olojede A.O., Sanni A.I., Banwo K. (2020). Effect of legume addition on the physiochemical and sensorial attributes of sorghum-based sourdough bread. LWT Food Sci. Technol..

[34] Zhu F. (2018). Chemical composition and food uses of teff (*Eragrostis tef*). Food Chem..

[35] Alvarez-Jubete L., Arendt E.K., Gallagher E. (2009). Nutritive value and chemical composition of pseudocereals as gluten-free ingredients. Int. J. Food Sci. Nutr..

[36] Arendt E.K., Zannini E. (2013). Amaranth. Cereal Grains for the Food and Beverage Industries.

[37] Lu H., Guo L., Zhang L., Xie C., Li W., Gu B., Li K. (2020). Study on quality characteristics of cassava flour and cassava flour short biscuits. Food Sci. Nutr..

[38] Skendi A., Mouselemidou P., Papageorgiou M., Papastergiadis E. (2018). Effect of acorn meal-water combinations on technological properties and fine structure of gluten-free bread. Food Chem..

[39] Angelov A., Yaneva-Marinova T., Gotcheva V. (2018). Oats as a matrix of choice for developing fermented functional beverages. J. Food Sci. Technol..

[40] Verma D.K., Srivastav P.P. (2017). Proximate composition, mineral content and fatty acids analyses of aromatic and non-aromatic Indian rice. Rice Sci..

[41] Gwirtz J.A., Garcia-Casal M.N. (2013). Processing maize flour and corn meal food products. Ann. N. Y. Acad. Sci..

[42] Rocha J.M., Malcata F.X. (2016). Microbial ecology dynamics in Portuguese broa sourdough. J. Food Qual..

[43] Rocha J.M., Malcata F.X. (2016). Behavior of the complex micro-ecology in maize and rye flour and mother-dough for broa throughout storage. J. Food Qual..

[44] Rocha J.M., Malcata F.X. (2012). Microbiological profile of maize and rye flours, and sourdough used for the manufacture of traditional Portuguese bread. Food Microbiol..

[45] Rocha J.M., Malcata F.X. (1999). On the microbiological profile of traditional Portuguese sourdough. J. Food Prot..

[46] Dereje N., Bekele G., Nigatu Y., Worku Y., Holland R.P. (2019). Glycemic index and load of selected Ethiopian foods: An experimental study. J. Diabetes Res..

[47] Gebru Y.A., Hyun-II J., Young-Soo K., Myung-Kon K., Kwang-Pyo K. (2019). Variations in amino acid and protein profiles in white versus brown teff (*Eragrostis tef*) seeds, and effect of extraction methods on protein yields. Foods.

[48] Fliedel G., Marti A., Thiebaut S. (1996). Caracterisation et valorization du sorgho. Bibliogr. CIRAD.

[49] Arendt E.K., Zannini E. (2013). 10-Teff Cereal Grains for the Food and Beverage Industries.

[50] Dingeo C., Difonzo G., Paradiso V.M., Rizzello C.G., Pontonio E. (2020). Teff type-I sourdough to produce gluten-free muffin. Microorganisms.

[51] Bultosa G., Hall A.N., Taylor J.R.N. (2002). Physico-chemical characterization of grain teff (*Eragrostis tef* (Zucc.) Trotter) starch. Starch.

[52] Campo E., del Arco L., Urtasun L., Oria R., Ferrer-Mairal A. (2016). Impact of sourdough on sensory properties and consumers preference of gluten-free breads enriched with teff flour. J. Cereal Sci..

[53] Wolter A., Hager A.S., Zannini E., Galle S., Gänzle M.G., Waters D.M., Arendt E.K. (2014). Evaluation of exopolysaccharide producing *Weissella cibaria* MG1 strain for the production of sourdough from various flours. Food Microbiol..

[54] Barretto R., Buenavista R.M., Rivera J.L., Wang S., Vara Prasad P.V., Siliveru K. (2021). Teff (*Eragrostis tef*) processing, utilization and future opportunities: A review. Int. J. Food Sci. Technol..

[55] Moroni A.V., Arendt E.K., dal Bello F. (2011). Biodiversity of lactic acid bacteria and yeasts in spontaneously-fermented buckwheat and teff sourdoughs. Food Microbiol..

[56] Harth H., van Kerrebroeck S., Vuyst L. (2018). Impact of process conditions on the microbial community dynamics and metabolite production kinetics of teff sourdough fermentations under bakery and laboratory conditions. Food Sci. Nutr..

[57] De Vuyst L., van Kerrebroeck S., Harth H., Huys G., Daniel H.-M., Weckx S. (2014). Microbial ecology of sourdough fermentations: Diverse or uniform?. Food Microbiol..

[58] Ogunsakin O.A., Banwo K., Ogunremi O.R., Sanni A.I. (2015). Microbiological and physicochemical properties of sourdough bread from sorghum flour. Int. Food Res. J..

[59] Awika J.M., Rooney L.W. (2004). Sorghum phytochemicals and their potential impact on human health. Phytochemistry.

[60] Dykes L., Rooney L.W. (2007). Phenolic compounds in cereal grains and their health benefits. Cereal Foods World.

[61] AACC International (2010). AACC Approved Methods of Analysis.

[62] Petkova M., Stefanova P., Gotcheva V., Kuzmanova I., Angelov A. (2020). Microbiological and physicochemical characterization of traditional Bulgarian sourdoughs and screening of lactic acid bacteria for amylolytic activity. J. Chem. Technol. Metal..

[63] Petkova M., Stefanova P., Gotcheva V., Angelov A. (2021). Isolation and characterisation of lactic acid bacteria and yeasts from typical Bulgarian sourdoughs. Microorganisms.

[64] Vasileva I., Denkova R., Chochkov R., Petkova N., Teneva D., Denkova Z., Desev T., Denev P., Slavov A. (2018). Effect of lavender (*Lavandula angustifolia*) and melissa (*Melissa officinalis*) waste on quality and shelf life of bread. Food Chem..

[65] ICC (2021). Standard Methods of the International Association for Cereal Science and Technology.

[66] Novotni D., Čukelj N., Smerdel B., Bituh M., Dujmić F., Ćurić D. (2012). Glycemic index and firming kinetics of partially baked frozen gluten-free bread with sourdough. J. Cereal Sci..

[67] Kim W.M., Gyu-Hee Lee G.H. (2015). Comparison of imported wheat flour bread making properties and korean wheat flour bread making properties made by various bread making methods. J. Korean Soc. Food Sci. Nutr..

[68] Lönner C., Preve-Akesson K. (1989). Effects of lactic acid bacteria on the properties of sour dough bread. Food Microbiol..

[69] Zlateva D., Chochkov R. (2019). Effect of *Spirulina platensis* on the crumb firming of wheat bread during storage. Ukr. Food J..

[70] (2017). Sensory Analysis—Methodology—General Guidance.

[71] Bower J.A. (1998). Statistics for food science V: ANOVA and multiple comparisons. Nutr. Food Sci..

[72] Puncha-arnon S., Uttapap D. (2013). Rice starch vs. rice flour: Differences in their properties when modified by heat–moisture treatment. Carbohydr. Polym..

[73] Gebru Y.A., Sbhatu D.B., Kim K.P. (2020). Nutritional composition and health benefits of teff (*Eragrostis tef* (Zucc.) Trotter). J. Food Qual..

[74] Di Monaco R., Torrieri E., Pepe O., Masi P., Cavella S. (2015). Effect of sourdough with exopolysaccharide (EPS)-producing lactic acid bacteria (LAB) on sensory quality of bread during shelf life. Food Bioproc. Technol..

[75] Antognoni F., Mandrioli R., Bordoni A., di Nunzio M., Viadel B., Gallego E., Laure D. (2017). Integrated evaluation of the potential health benefits of einkorn-based breads. Nutrients.

[76] Ua-Arak T., Jakob F., Vogel R.F. (2017). Influence of levan-producing acetic acid bacteria on buckwheat-sourdough breads. Food Microbiol..

[77] Hadaegh H., Seyyedain Ardabili S., Tajabadi Ebrahimi M., Chamani M., Azizi Nezhad R. (2017). The impact of different lactic acid bacteria sourdoughs on the quality characteristics of toast bread. J. Food Qual..

[78] Topuzova Y.A., Karadzhov G.I., Chonova V.M. (2012). Basic raw materials used for production of gluten-free bakery and confectionery products. Sci. Works UFT.

[79] Alaunyte I., Stojceska V., Plunkett A., Ainsworth P., Derbyshire E. (2012). Improving the quality of nutrient-rich Teff (*Eragrostis tef*) breads by combination of enzymes in straight dough and sourdough breadmaking. J. Cereal Sci..

[80] Hager A.S., Arendt E.K. (2013). Influence of hydroxypropylmethylcellulose (HPMC), xanthan gum and their combination on loaf specific volume, crumb hardness and crumb grain characteristics of gluten-free breads based on rice, maize, teff and buckwheat. Food Hydrocol..

[81] Neela S., Fanta S.W. (2020). Injera (An ethnic, traditional staple food of Ethiopia): A review on traditional practice to scientific developments. J. Ethn. Food.

[82] Arendt E.K., Morrissey A., Moore M.M., dal Bello F., Arendt E.K., dal Bello F. (2008). Gluten-Free Breads. Gluten-Free Cereal Products and Beverages.

[83] Galassi E., Taddei F., Ciccoritti R., Nocente F., Gazza L. (2020). Biochemical and technological characterization of two C4 gluten-free cereals: *Sorghum bicolor* and *Eragrostis tef*. Cereal Chem..

[84] Arendt E.K., dal Bello F., Hamaker B.R. (2008). Functional Cereal Products for Those with Gluten Intolerance. Technology of Functional Cereal Products.

[85] Duodu K.G., Taylor J.R.N., Cauvain S.P. (2012). The Quality of Breads Made with Non-Wheat Flours. Breadmaking.

[86] Marti A., Marengo M., Bonomi F., Casiraghi M.C., Franzetti L., Pagani M.A., Iametti S. (2017). Molecular features of fermented teff flour relate to its suitability for the production of enriched gluten-free bread. LWT.

[87] Yilmaz H.O., Arslan M. (2018). Teff: Nutritional compounds and effects on human health. Acta Sci. Med. Sci..

[88] Olojede A.O., Sannic A.I., Banwo K. (2020). Rheological, textural and nutritional properties of gluten-free sourdough made with functionally important lactic acid bacteria and yeast from Nigerian sorghum. LWT Food Sci. Technol..

[89] Rico D., Ronda F., Villanueva M., Perez Montero C., Martin-Diana A.B. (2019). Development of healthy gluten-free crackers from white and brown teff (*Eragrostis tef Zucc.*) flours. Heliyon.

[90] Chanapamokkhot H., Thongngam M. (2007). The chemical and physico-chemical properties of sorghum starch and flour. Kasetsart J..

[91] Pandya T.S., Srinivasan R. (2012). Effect of hammer mill retention screen size on fiber separation from corn flour using the Elusieve process. Ind. Crops Prod..

[92] Zannini E., Pontonio E., Waters D.M., Arendt E.K. (2012). Applications of microbial fermentations for production of gluten-free products and perspectives. Appl. Microbiol. Biotechnol..

[93] Ćuric D., Novotni D., Tusak D., Bauman I., Gabric D. (2007). Gluten-free bread production by the corn meal and soybean flour extruded blend usage. Agric. Consp. Sci..

[94] Wolter A., Hager A.S., Zannini E., Czerny M., Arendt E.K. (2014). Impact of sourdough fermented with *Lactobacillus plantarum* FST 1.7 on baking and sensory properties of gluten-free breads. Eur. Food Res. Technol..

[95] Nami Y., Gharekhani M., Aalami M., Hejazi M.A. (2019). Lactobacillus-fermented sourdoughs improve the quality of gluten-free bread made from pearl millet flour. J. Food Sci. Technol..

[96] Edema M.O., Emmambux M.N., Taylor J.R.N. (2013). Improvement of fonio dough properties through starch modification by sourdough fermentation. Starch.

[97] Schober T.J., Bean S.R., Boyle D.L. (2007). Gluten-free sorghum bread improved by sourdough fermentation: Biochemical, rheological, and microstructural background. J. Agric. Food Chem..

[98] Sahin A.W., Wiertz J., Arendt E.K. (2020). Evaluation of a new method to determine the water addition level in gluten-free bread systems. J. Cereal Sci..

[99] Tafti A.G., Peighardoust S.H., Behnam F., Bahrami A., Aghagholizadeh R., Ghamari M., Rafat S.A. (2013). Effects of spray-dried sourdough on flour characteristics and rheological properties of dough. Czech J. Food Sci..

[100] Moore M.M., dal Bello F., Arendt E.K. (2008). Sourdough fermented by *Lactobacillusplantarum* FST 1.7 improves the quality and shelf life of gluten-free bread. Eur. Food Res. Technol..

[101] Wolter A., Hager A.S., Zannini E., Czerny M., Arendt E.K. (2014). Influence of dextran-producing *Weissella cibaria* on baking properties and sensory profile of gluten-free and wheat breads. Int. J. Food Microbiol..

[102] Axel C., Rocker B., Brosnan B., Zannini E., Furey A., Coffey A., Arendt E.K. (2015). Application of *Lactobacillus amylovorus* DSM19280 in gluten-free sourdough bread to improve the microbial shelf life. Food Microbiol..

[103] Di Cagno R., Rizzello G.C., de Angelis M., Cassone A., Giuliani G., Benedusi A., Limitone A., Surico R.F., Gobbetti M. (2008). Use of selected sourdough strains of *Lactobacillus* for removing gluten and enhancing the nutritional properties of gluten-free bread. J. Food Prot..

[104] Falade A.T., Emmambux M.N., Buys E.M., Taylor J.R.N. (2014). Improvement of maize bread quality through modification of dough, rheological properties by lactic acid bacteria fermentation. J. Cereal Sci..

[105] Moghaddam M.F.T., Jalali H., Nafchi A.M., Nouri L. (2020). Evaluating the effects of lactic acid bacteria and olive leaf extract on the quality of gluten-free bread. Gene Reports.

[106] Corsetti A., Settanni L. (2007). Lactobacilli in sourdough fermentation. Food Res. Int..

[107] Hansen A., Schieberle P. (2005). Generation of aroma compounds during sourdough fermentation: Applied and fundamental aspects. Trends Food Sci. Technol..

[108] Bourne M.C. (1978). Texture profile analysis. Food Technol..

[109] Poutanen K., Flander L., Katina K. (2009). Sourdough and cereal fermentation in a nutritional perspective. Food Microbiol..

[110] Tieking M., Gänzle M.G. (2005). Exopolysaccharides from cereal-associated lactobacilli. Trends Food Sci. Technol..

[111] Belz M.C.E., Axel C., Arendt E.K., Lynch K.M., Brosnan B., Sheehan E.M., Coffey A., Zannini E. (2019). Improvement of taste and shelf life of yeasted low-salt bread containing functional sourdoughs using *Lactobacillus amylovorus* DSM 19280 and *Weisella cibaria* MG1. Int. J. Food Microbiol..

